# Treatment and Outcomes of Unifocal and Multifocal Osseous Pelvic Langerhans Cell Histiocytosis Lesions in a Pediatric Population

**DOI:** 10.7759/cureus.28470

**Published:** 2022-08-27

**Authors:** Parker Mitchell, Ekene U Ezeokoli, Neritan Borici, Eva Schleh, Nicole Montgomery

**Affiliations:** 1 Orthopaedic Surgery, Baylor College of Medicine, Houston, USA; 2 Orthopaedic Surgery, Texas Children's Hospital, Houston, USA; 3 Orthopaedic Surgery, Oakland University William Beaumont School of Medicine, Rochester, USA; 4 Orthopaedic Surgery, University of Medicine, European Hospital Villa Maria, Tirana, ALB; 5 Orthopaedic Surgery, Jacobs School of Medicine and Biomedical Sciences, University at Buffalo, Buffalo, USA

**Keywords:** chemotherapy, histiocytosis, lesion, hip, pelvis, lch, langerhans

## Abstract

Introduction

Langerhans cell histiocytosis (LCH) is a rare, clonal disorder characterized by proliferation and tissue infiltration by myeloid dendritic cells, most commonly occurring in pediatric populations. It often manifests as skeletal lesions with possible pelvic involvement. Few studies have characterized and reviewed outcomes after treatment of isolated pelvic LCH lesions.

Methods

A retrospective single-institution review was conducted on diagnoses of patients younger than 18 with a diagnosis of unifocal or multifocal skeletal LCH lesions involving the pelvis. Clinical presentations, lesion sites, focal classification, radiographic findings, treatments, complications, and recurrence rates were reviewed.

Results

Twenty patients had unifocal or multifocal LCH pelvic lesions (11 males, nine females). The median age at diagnosis was 3.5 years (0.8-21.6). Eight cases (40%) involved unifocal lesions, and twelve (60%) involved multifocal lesions, with the most common associated skeletal disease occurring at the ilium. 100% of cases had a lytic bone lesion with no pathologic fractures. All cases were treated nonoperatively with chemotherapy medications, corticosteroids, or observation alone. 75% of cases were treated with chemotherapy with a 100% resolution rate. The median length of follow-up was 4.5 years (0.4-16.7).

Conclusion

Our study found that chemotherapy alone or chemotherapy with corticosteroid supplementation are appropriate options for unifocal pelvic LCH lesions. In contrast, pelvic lesions that are part of a multifocal presentation may be managed adequately with varied chemotherapy regimens. Corticosteroid therapy and observation alone may also be reasonable for a single organ system, multifocal, skeletal lesions that are anatomically accessible for biopsy and small in number or size.

## Introduction

Langerhans Cell Histiocytosis (LCH) is a rare, neoplastic histiocytic disorder that most commonly affects bones and skin; however, it can also involve the lungs, pituitary gland, liver, spleen, central nervous system, lymph nodes, or other organs. LCH is considerably more common in the pediatric population, especially in white males, and is characterized by clonal proliferation of Langerhans dendritic cells [1]. Although the neoplastic cells of LCH resemble dendritic Langerhans cells in the skin and mucosa, the CD1a+ and Langerin+ neoplastic cells of LCH are found in bone, and visceral organs are derived from myeloid dendritic cells.

Initial presentation can range from isolated and unifocal skeletal lesions to multisystemic disseminated involvement, depending on the progression of the disease. In pediatric populations, LCH is limited to one organ system in approximately 55% of cases, with the remainder presenting with multisystemic disease [2]. The multisystem disease is most often seen in children under three years of age, while single-organ involvement is more common in older children and adults. The Histiocyte society classifies the disease as single-system single-site (SS-s), single-system multiple-site (SS-m), and multisystem (MS) [3]. Complete remission is sometimes possible without treatment, depending on the presentation, but some cases may have treatment-resistant resistance, swift progression, post-disease complications, or even death [4-6]. SS-s usually have a better prognosis with more conservative treatment, while MS requires more aggressive management and is more likely to have a less desirable outcome.

Bone involvement is one of the most common findings of LCH. Although some bone lesions are asymptomatic, pain with accompanying skeletal lesions and raised, soft, tender spots are also common initial presentations [7]. LCH can involve any bone in the body, but the most common sites vary with age. Children's most frequent sites are the skull, femur, rib, vertebra, and humerus [8]. The main sites of bone involvement in adults are the jaw, skull, pelvis, vertebra, extremities, and rib [9]. While the pelvis is not an uncommon site of LCH lytic lesions in adult studies, few studies have characterized and reviewed the eventual outcomes after treatment at these sites, especially in pediatric populations. The few studies looking at isolated pelvic lesions were all case reports in an older patient population or investigations characterizing radiographic findings for differential diagnosis [10-17]. The incidence and sequential investigation of outcomes of this rare disorder at the pelvic site are poorly defined and followed.

This study's primary goal includes (1) clinical and radiographic characterization of a series of unifocal and multifocal LCH lesions involving the pelvis and (2) determining success and recurrence rates with different treatment modalities in a pediatric population at a tertiary children's hospital.

## Materials and methods

We obtained approval from the Baylor Institutional Review Board (H-45616) for a retrospective review of patients under 18 years old diagnosed with LCH at a significant, level 1 children's hospital before June 1, 2021. Patients were captured from February 22, 2005, to June 1, 2021. The main inclusion criteria included patients with a unifocal or multifocal skeletal lesion. After inclusion criteria were met, patients without a pelvic lesion were excluded. Additional exclusion criteria included the involvement of the bone marrow and multisystemic LCH disease, which included patients with visceral involvement, concurrent malignant diagnoses, and lack of data. Twenty patients met all criteria to be included in the study.

All patients had a skeletal survey and a positron emission tomography (PET) scan. The diagnosis was confirmed through biopsy and histology via a positive CD1a, CD207 (Langerin), or S100 immunoassay in all cases, except for Case 10, as no test was documented. Clinical presentations, lesion sites, additional skeletal lesions, biopsy site, focal classification, radiographic findings, lesion size, treatments, complications, recurrence rates, and lengths of follow-up, if present, were abstracted from the chart. We also determined whether the associated skeletal lesion was diagnosed at the initial consultation and which intervention was used in clinical care.

Data analysis

All statistics were descriptive and were reported as counts with percentages or means with range values or where applicable.

## Results

Demographics

In our study, there were 686 patients diagnosed with Langerhans cell histiocytosis during the study period between 2009-2021. Twenty patients were found to have unifocal or multifocal LCH lesions involving the pelvis. There were 11 males and nine females identified. The median age at diagnosis was 3.5 years (0.8-21.6) (Table 1). The most common reasons for exclusion were multisystemic cases or bone marrow involvement.

**Table 1:** Demographics and Characteristics M – male; F–female; N/A – not available or documented

Case	Age at diagnosis/Sex	Presentation	Lesion Site	Other skeletal lesion(s)	Classification	Biopsy site(s)
1	2.3/F	Restricted range of motion, limitation	Ilium	N/A	Unifocal	Hip
2	8.2/M	Upper leg pain	Sacrum	Femur, sternum	Multifocal	Femur
3	15.4/F	Sided weakness, dizziness, fatigue	Ischium	Vertebrae	Multifocal	Vertebrae
4	3.2/F	Painless limping	Ilium	N/A	Unifocal	Ilium
5	1.7/F	Painful limping	Ilium	N/A	Unifocal	Ilium
6	0.9/M	Appetite change, fever, irritability, rash	Ilium	Clavicle, rib	Multifocal	None
7	3.9/M	Limping, fatigue, sweating	Ilium	Left parietal	Multifocal	Ilium
8	3.0/F	Limping 8-9 weeks	Ilium	Vertebrae, femur, sacrum	Multifocal	Ilium
9	0.8/M	Leg pelvic pain	Ilium	Mediastinum	Multifocal	Acetabulum
10	10.5/M	Back pain	Acetabulum	Vertebrae, skull	Multifocal	Vertebrae
11	5.4/F	Headaches, nausea	Pubis, acetabulum	N/A	Unifocal	Acetabulum
12	21.6/M	Scalp mass	Bilateral ilium, pubis	Femur, rib, skull	Multifocal	Skull
13	13.2/M	Hip pain	Ischium	N/A	Unifocal	Ischium
14	8.6/F	Leg pain	Ilium	N/A	Unifocal	Ilium
15	2.1/M	Limping, leg mass	Ilium	femur, rib, humerus, metacarpal	Multifocal	Femur
16	1.8/M	Fever, rhinorrhea	Ischium	skull	Multifocal	Skull
17	11.7/M	Arm pain	Sacrum	clavicle, rib, vertebra	Multifocal	Clavicle
18	11.3/F	Hip pain, limping	Ilium	N/A	Unifocal	Ilium
19	1.1/M	Scalp rash	Ilium	Pelvis, femur, parietal bone	Unifocal	Skin
20	1.7/F	Hip pain	Ilium	Pelvis, femur, parietal bone	Multifocal	Ilium

Characterization

**Figure 1:**
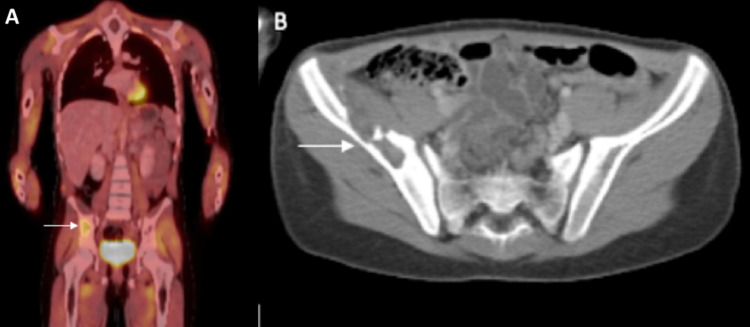
8-year-old female presenting with hip and lower extremity pain (Case 14). (A) AP PET/CT demonstrating hyperintensity of the right ilium. (B) Axial CT demonstrating an osteolytic lesion at the right ilium.

Of the 20 pelvic LCH cases identified, there were 8 (40%) unifocal lesions and 12 (60%) multifocal lesions, with the most common associated skeletal lesion occurring at the ilium (13 cases, 60%). Other sites included ischium (3 cases, 15%), acetabulum (2 cases, 10%), and sacrum (2 cases, 10%). The most common multifocal lesion site occurred at the ilium (7 cases, 58.3%), along with sacral (2 cases, 16.7%), ischial (2 cases, 16.7%), and acetabular (1 case, 8.3%) lesions. Of the eight unifocal lesions, the ilium was the most common site (6 cases, 75%). Case 12 included bilateral ilium and pubis involvement, and Case 11 included acetabular and pubis lesions. The ileum was typically chosen as a biopsy site in unifocal cases (4 cases, 50%). The most common radiographic finding was a lytic bone lesion (100% of cases, Figure 1). Measurements of pelvic lesions were not consistently assessed radiographically. The most common clinical presentations associated with pelvic lesions were pain and limping with lower extremity or hip pain occurring in 8 patients and limping in 7 patients with pelvic lesions. Other bony lesions primarily included the femur (5 cases, 25%), vertebrae (4 cases, 20%), ribs or mediastinum (6 cases, 30%), and skull (4 cases, 20%).

Treatments and outcomes

No patients with pelvic LCH had their lesions resected or debrided surgically. All cases were treated nonoperatively either exclusively or in tandem with chemotherapy medications, corticosteroids, or observation alone. Most cases were treated with vinblastine and prednisone (11 cases, 55%). Other treatment options included cytarabine (7 cases, 35%) or no treatment (3 cases, 15%). Two cases were eventually transitioned to cladribine (Case 1 and 8). Case 1 was transitioned from vinblastine and prednisone to cladribine due to a history of neutropenia with prior chemotherapy and LCH-S-98 study protocol. Case 8 was transitioned to cladribine due to complaints of bilateral heel and feet pain with mild foot drop secondary to vinblastine. Three chemotherapy regimens switched to clofarabine (Case 10, 12, 17) due to new skeletal lesions or increased size of lesions. Of the 8 unifocal cases, 5 cases were treated with vinblastine and prednisone, 2 cases had no treatment, and 1 case was treated with steroid injection alone. One patient had a documented disease recurrence. The recurring case (Case 13) included a unifocal lesion of the ischium that returned as cervical adenopathy. The initial ischial lesion was treated solely with triamcinolone acetonide injections, and the recurrent adenopathy was treated with hydroxyurea. The median length of follow-up was 4.5 years (0.4-16.7 years) (Table 2). There was no mortality in this cohort. No complications from treatment were recorded.

**Table 2:** Radiology, Histology, and Outcomes

Case	Radiographic findings	Size of the lesion (on imaging)	Immune Findings	Treatment	Recurrence frequency	Total f/u (years)
1	Lytic lesion (expansile with edema)	2.8x2.5x2.1 cm	CD207+	Vinblastine, prednisone, cladribine	0	6.32
2	Lytic lesion	N/A	CD1a+, S100+	Vinblastine, prednisone	0	3.05
3	Lytic lesion	0.5x0.6x0.9 mm	CD1a+, CD207+, S100+	None	0	0.36
4	Osteolytic lesion	4.2x1.6 cm	CD1a+, S100+	Vinblastine, prednisone	0	9.55
5	Lytic lesion	N/A	CD1a+	None	0	0.52
6	Lytic lesion	N/A	CD1a+​​, S100+	Cytarabine	0	1.56
7	Lytic lesion	N/A	CD1a+	Cytarabine	0	3.14
8	Lytic lesion (slight sclerosis)	2.0x1.5x2.4 cm	CD1a+, CD207+	Vinblastine, and prednisone, were switched to cladribine. Continued 6-mercaptopurine and methotrexate for maintenance therapy	0	10.97
9	Lytic lesion	2.7cm	CD207+	Vinblastine, cytarabine	0	16.13
10	Lytic lesion	N/A	N/A	Vinblastine, prednisone, cytarabine clofarabine	0	5.86
11	Lytic lesion	3.5x3.3x2.1 cm	CD207+	Vinblastine, prednisone	0	11.77
12	Lytic lesion (sclerotic)	6.7mm x 2.6mm	CD1a+, CD207+, S100+	Cytarabine + zoledronic acid, switched to clofarabine, after 3 months	0	0.88
13	Lytic lesion	N/A	CD1a+, S100+	Steroid injection	1	1.42
14	Lytic lesion	N/A	CD1a+	Vinblastine, prednisone	0	1.21
15	Lytic lesion	2x0.8x0.2 cm	CD1a+, CD207+, S100+	Cytarabine	0	8.78
16	Lytic lesion	N/A	CD207+	Vincristine	0	11.41
17	Lytic lesion	2cm	CD207+	Cytarabine switched to Clofarabine	0	6.77
18	Lytic lesion (cortical disruption)	2.2x2.1x1.8 cm	CD1a+, CD207+	None	0	0.83
19	Lytic lesion	N/A	CD1a+	Vinblastine, prednisone	0	1.81
20	Lytic lesion	N/A	CD1a+	Vinblastine, prednisone	0	16.67

## Discussion

Langerhans cell histiocytosis is a rare disease expressed as abnormal histiocytic proliferation, often occurring with skeletal lesions or skin involvement. In pediatric patients, treatment is usually adapted to presentation depending on disease progression and organ involvement. Isolated lesions have different treatment options, while multisystemic or disseminated disease usually requires chemotherapy [8]. Some studies have even demonstrated that minimally-invasive radiofrequency ablation may support safe and effective treatment for unifocal LCH bone lesions [17]. LCH presentations may not be benign, with disseminated disease associated with increased mortality and recurrence rates [18-21].

We only identified and analyzed unifocal (SS-s) or multifocal (SS-m) pelvic lesions; therefore, no patients displayed disseminated or visceral disease signs. Of the 686 patients initially queried for a diagnosis of LCH, twenty included lesions involving the pelvis (2.9%), with 8 being unifocal pelvic lesions (1.2%). All unifocal lesions were treated via vinblastine, corticosteroids, corticosteroids alone, or observation. Current literature suggests that resection of the skeletal lesion and steroid injection lead to positive outcomes in unifocal LCH lesions [22-24]. In various cases, resection and steroid injection can be used as an adjunct to chemotherapy or radiotherapy depending on the lesion site or associated complications [18,25]. Other studies have shown that skeletal, unifocal LCH lesions do not likely require aggressive surgical or medical management. A recent study by Rivera et al. [26] further demonstrated that biopsy alone confirms the diagnosis of unifocal osseous LCH and can rapidly resolve symptoms.

In comparison, all multifocal lesions in our study were treated with various chemotherapy regimens, primarily vinblastine and prednisone, cytarabine, cladribine, clofarabine, or observation alone of additional lesion quantity or location. Depending on the disease progression and location or the number of additional lesions, treatment was varied in a case-by-case manner. All patients experienced successful outcomes in our cohort regardless of treatment, except Case 13, who displayed a recurrent LCH lymphadenopathy event. However, three patients had under a year of follow-up.

Due to the location of the lesions in our study, most of the cases presented with acute lower back, hip, or leg pain and limping on the affected side. Despite the location of lesions in the cohort and several primary presentations, including acetabular involvement and deformity, there was no documented flank pain, constipation, buttocks pain, restriction of hip movement, associated fractures, functional impairment, or other complications expected from pathologies in the pelvis or acetabular joint.

Currently, no available case reports discuss longitudinal outcomes in pediatric patients with isolated, pathologic pelvic fractures. Furthermore, there is limited literature on pelvic LCH lesions in pediatric patients. Previous studies have investigated radiographic findings and imaging factors to differentiate diagnoses [10-14]; however, few studies thoroughly investigate LCH pelvic lesions through management, outcomes after treatment, or subsequent follow-up assessment. As LCH is often highly treatable, with many patients exhibiting favorable outcomes, timely diagnosis and management are essential, especially in patients with uncomplicated, superficial bony lesions. Although treatment can vary on a case-by-case basis, varying options in adults with isolated skeletal LCH have included curettage and corticosteroid injections [2,15,27] with or without supplemental radiation [28], and non-operative observation [29].

Limitations

A weakness of our study is its retrospective nature which relies on a chart review of available imaging and documentation. This retrospective case series was limited by the infrequency of LCH pelvic lesion presentations and would benefit from a higher-powered randomized trial to compare treatment options and outcomes of LCH lesions involving the pelvis. This was a single-institution study. Furthermore, there were only 20 patients that fit the criteria for our series. Though most literature reports of LCH are case reports, our study cohort is still of a relatively low quantity. Only 8 of our patients had a unifocal and isolated pelvic lesion, and four had under one year of follow-up. While we found an association of LCH pelvic lesion resolution with non-operatively treated methods, our study did not evaluate pelvic lesions treated operatively, which might reveal different rates of resolution and recurrence.

## Conclusions

Our study found that chemotherapy regimens alone or with adjuvant corticosteroid supplementation are an appropriate option for unifocal pelvic LCH lesions. Multifocal pelvic lesions may be managed adequately with varied chemotherapy regimens, and surgical resection was not demonstrated as a suggested treatment option for LCH lesions involving the pelvis. Steroid therapy or observation alone may also be reasonable for pediatric, single-system, multifocal, skeletal lesions that are small in number and size, in contrast with a complete chemotherapy regimen and its associated adverse effects.
